# Prevalence of depression, anxiety and stress disorders among medical students in Alexandria Faculty of Medicine during COVID-19 pandemic

**DOI:** 10.1192/bjo.2021.68

**Published:** 2021-06-18

**Authors:** Hesham Adel Sheshtawy, Sarah Hemead, Ahmed Shaheen, Nour Shaheen, Ehab Elrewany, Hanan M. Hemead

**Affiliations:** 1 Alexandria Faculty of Medicine; 2 High Institute of Public Health, Alexandria University

## Abstract

**Aims:**

We aimed to assess the impact of the current pandemic on the mental well-being of undergraduate medical students of Alexandria Faculty of Medicine, Egypt.

**Method:**

We designed a structured anonymous online questionnaire and encourage students to fill it in on social platforms. The questionnaire is composed of seven parts, each one includes multiple choice questions aimed to measure the impact of the pandemic on different aspect of daily activities namely: academic performance, social and family relationship, eating and smoking habits, sleep pattern, physical activity and the Depression, anxiety and stress scale (DASS-21). The last part was an open question where participants can state their comments about the experiences during the quarantine and how they affected their mental health.

**Result:**

A total of 1181 students from the six academic grades responded. Females and students in the third academic year showed the highest prevalence of depression, anxiety and stress. Overall, most respondents reported that the current pandemic had negative impacts on their academic performance (71%) and social relationship (67.5 %). The majority of the students stated that they became less physically active (74.6%) and 52.2% experienced a weight gain. Despite that 60% of the studied population rated their sleeping quality as ‘’very good’’ and ‘’fairly good’’, 45.3% and 39.6% suffered from increased sleeping hours and disturbed sleep respectively. Based on students’ responses of the DASS-21, over half of the participants (62.2%) were experiencing moderate to extremely severe stress and over 33% were consistent with symptoms of extremely severe anxiety. In respect of depression, nearly half of the sample (46.4%) can be described as having extremely severe depression according to the cut-off points of the DASS-21.

**Conclusion:**

The current pandemic has increased the challenges and burdens on undergraduate medical students. These impacts can be more profound in developing countries such as Egypt. The levels of psychiatric symptoms are alarming compared to previous local and international studies. These unprecedented consequences should be addressed promptly through students’ counselling and psychiatric assistance. To date, this is the largest psychiatric and survey-based study conducted on Alexandria Faculty of Medicine.

Financial disclosure: The study was not funded by any organization, the authors did not receive any financial aids.

